# Insights and inferences about integron evolution from genomic data

**DOI:** 10.1186/1471-2164-9-261

**Published:** 2008-05-31

**Authors:** Diana R Nemergut, Michael S Robeson, Robert F Kysela, Andrew P Martin, Steven K Schmidt, Rob Knight

**Affiliations:** 1 Institute of Arctic and Alpine Research, University of Colorado, Boulder, Colorado, USA; 2 Environmental Studies Program, University of Colorado, Boulder, Colorado, USA; 3 Department of Ecology and Evolutionary Biology, University of Colorado, Boulder, Colorado, USA; 4 Department of Chemistry and Biochemistry, University of Colorado, Boulder, Colorado, USA

## Abstract

**Background:**

Integrons are mechanisms that facilitate horizontal gene transfer, allowing bacteria to integrate and express foreign DNA. These are important in the exchange of antibiotic resistance determinants, but can also transfer a diverse suite of genes unrelated to pathogenicity. Here, we provide a systematic analysis of the distribution and diversity of integron *intI *genes and integron-containing bacteria.

**Results:**

We found integrons in 103 different pathogenic and non-pathogenic bacteria, in six major phyla. Integrons were widely scattered, and their presence was not confined to specific clades within bacterial orders. Nearly 1/3 of the *intI *genes that we identified were pseudogenes, containing either an internal stop codon or a frameshift mutation that would render the protein product non-functional. Additionally, 20% of bacteria contained more than one integrase gene. dN/dS ratios revealed mutational hotspots in clades of *Vibrio *and *Shewanella intI *genes. Finally, we characterized the gene cassettes associated with integrons in *Methylobacillus flagellatus *KT and *Dechloromonas aromatica *RCB, and found a heavy metal efflux gene as well as genes involved in protein folding and stability.

**Conclusion:**

Our analysis suggests that the present distribution of integrons is due to multiple losses and gene transfer events. While, in some cases, the ability to integrate and excise foreign DNA may be selectively advantageous, the gain, loss, or rearrangment of gene cassettes could also be deleterious, selecting against functional integrases. Thus, such a high fraction of pseudogenes may suggest that the selective impact of integrons on genomes is variable, oscillating between beneficial and deleterious, possibly depending on environmental conditions.

## Background

Horizontal gene transfer (HGT) is effected through some combination of the activity of mobile gene elements and/or recipient cellular enzyme systems. In the most general terms, the process of horizontal gene transfer requires several, distinct steps [1]. The mechanisms and controls over processes for physically transferring DNA into recipient cells (i.e., transformation, conjugation and transduction) have received significant attention in the literature. However, the ability of cells to replicate and express foreign DNA is also essential for the transferred genes to become prevalent in the population through natural selection [1]. Mobile gene elements can thus be important in promoting HGT because they can contain origins of replication (ORIs) and/or promoters to facilitate the replication and transcription of foreign DNA. ORIs and promoters vary widely both within and between different species of bacteria, and may be more likely to be recognized by the cellular machinery of closely related organisms than more distantly related species, limiting the phylogenetic distance over which certain mobile gene elements can be transferred. However, many cross-phyla [e.g., [2]] and even cross-domain [e.g., [3]] gene transfer events have been documented. Although broad host range plasmids [4,5] and phages [6] play important roles in these long distance transfers, questions remain about the potential role of other mobile elements.

Integrons [7], are gene elements which may also play an important role in the transfer of genes between distantly related lineages. Integrons facilitate the integration [8], excision [9], and rearrangement [10] of mobile genes which contain *attC *(also referred to as 59-be) recombination sites [8,11], called "gene cassettes" [12]. Once integrated, the gene cassette is then expressed from the P_c _promoter Integrons catalyze the integration of foreign genes into a DNA molecule that is already recognized by the native replication machinery (chromosome or plasmid), and under the control of a promoter that allows gene expression in the host. Thus, this mechanism permits organisms to sample from the diversity of gene cassettes in their local environment without the need for host recognition of foreign promoters or ORIs, and may therefore facilitate gene transfer among highly divergent groups of bacteria.

Integrons are classified according to the sequence of the IntI protein. The first integron discovered, the class 1 integron, is now known to be important in the dissemination of antibiotic resistance genes in both Gram-negative and Gram-positive bacteria [14]. These integrons are typically found on plasmids or transposons, which catalyze their own mobility. Class 1 integrons have been found in association with dozens of different resistance genes [reviewed in [14]], and have been found in environments ranging from hospitals to poultry litter [15,16]. Other types of integrons, including classes 2, 3, 9, and an unnamed class found on a *Vibrio salmonicida *plasmid, (GenBank accession number AJ277063) have been similarly found on mobile elements and in association with antibiotic resistance genes [17-20]. Collectively, these are sometimes called "mobile integrons" or "multi-resistance" integrons; however, because the genomic location and associated gene cassettes are not stable features of integrons, this nomenclature has been discouraged [21,22]. Indeed, integrons associated with antibiotic genes have been mobilized several times in the history of their evolution [23], leading to a major public health concern.

Genome sequencing projects [17,23,24] have revealed a wide variety of *intI *genes from phylogenetically diverse bacteria (no reports of integrons in the Eucarya or Archaea have been made to date), and a recent study demonstrates that they are present in ~10% of sequenced genomes [23]. Boucher et al. [23] used BLAST searches and identified integrons in the genomes of organisms from the Spirochaetes, Cyanobacteria, Chlorobi, Planctomycetes and the γ, β, δ, and ε Proteobacteria. Mazel [17] identified two major clades of *intI *genes, one arising from organisms living in soil/freshwater environments and the other found in marine bacteria. Cultivation-independent studies have revealed an enormous diversity of *intI *genes in a variety of environmental samples [25]. Likewise, a plethora of different types of gene cassettes, many having no obvious role in pathogenicity or resistance phenotypes, have been found associated with these integrase genes [e.g., [26-28]], and in environmental metagenomic samples [25,27,29,30]. In some cases, such as within species of the genus *Vibrio*, integrase genes are found in association with over 100 gene cassettes [31-33], suggesting that these elements play a major role in the evolution of these organisms. Thus, it has been hypothesized that their wide distribution and in some cases major genome load may make these elements important in the evolution of a diverse, environmentally-relevant suite of bacteria.

However, many fascinating questions remain about the nature of integrons. For example, why are these potentially adaptive elements found in some organisms but not in others? One major force in the evolution of integrons is thought to be mobility – through association with plasmids or transposons – and much evidence suggests that integrons themselves are transferred between bacterial lineages [23,25]. However, there is support for a good deal of vertical inheritance for integrons as well [23,25]. Here, we use genomic analyses to examine integron diversity and distribution. We also describe the types of gene cassettes found in association with two organisms, further emphasizing the diversity of genes that integrons can mobilize.

## Results and Discussion

### Which lineages contain integrons?

We used BLAST to search for *intI *genes in organisms whose whole genomes have been partially or completely sequenced, and to query sequences in the nr GenBank database. Although the promoter and *attI *recombination site are integral parts of both integron structure and function, we chose to broaden our definition and search for just the presence of integron integrase genes because IntI can also catalyze recombination events between the *attC *site and secondary sites [20,34,35]. These events occur at low frequencies, but may be important for bacterial evolution because they result in the insertion of a gene cassette that is flanked by a single recombination site, significantly lowering the possibility of IntI-mediated excision [35]. Thus, we use the terms "integron integrase gene" "*intI*", and "integron" interchangeably.

We identified a total of 103 different bacteria that contain integron integrase genes (the typically mobile integrons were excluded from this analysis but have been reviewed elsewhere [14]). We found *intI *genes in eighteen different bacterial orders within six divisions, including the Bacteroidetes/Chlorobi group, Chloroflexi, Cyanobacteria, Planctomycetes, Proteobacteria and Spirochaetes (Additional File 1), expanding on the diversity recovered from the most recent survey [23]. These phylogenetically diverse organisms are found in a variety of both oxic and anoxic environments, and their metabolisms range from heterotrophy to photoautotrophy. The diversity of integron-containing organisms suggests that either these elements are ancient, playing significant roles in shaping microbial genomes over long timescales, or that the evolutionary advantage of being able to catalyze integron-mediated gene transfer has led to the more recent, rapid dispersal of integrons among disparate bacterial lineages.

There are many biases associated with the selection of organisms for both laboratory work and for whole genome sequencing, so the lack of a particular type of organism or group of organisms in Additional File 1 should not be interpreted as evidence for the lack of an integron (with the exception of within specific, entirely sequenced genomes, see below). However, some divisions and subdivisions have been the targets of major sequencing efforts yet are notably absent from this table, including organisms within the Actinobacteria (37 complete genomes sequenced representing 3 orders), and the Firmicutes (103 complete genomes representing 7 orders). It is unknown whether integrons are missing from these lineages all together, or if they are merely absent from the specific organisms selected for sequencing projects. This uncertainty is especially interesting in light of the importance of Gram positive organisms as environmental reservoirs of class 1 integrons [15]. In addition, none of the 54 α-proteobacterial genomes, representing 6 orders, contain integrons, yet these elements are found in the γ, β, δ, and ε proteobacterial subdivisions (Additional File 1). To our knowledge, no type 1 integrons have been found in the α-proteobacteria.

Nearly one third of the *intI *genes that we uncovered are predicted to be non-functional, containing either a stop codon, a frameshift mutation, or a major insertion or deletion that would likely render them inactive (*, Additional File 1). The *Vibrios *appear to be an exception to this rule, as only 4% of *intI *genes from this group are pseudogenes. Another interesting feature of integron distribution is that more than one-fifth of all integron-containing lineages harbor more than one *intI *gene (Additional File 1). This is likely an underestimate of the fraction of organisms with two or more genes: fewer than half of the isolates in Additional File 1 are completely sequenced, and the remainder may contain additional integrase genes in the unsequenced regions. Again, the *Vibrios *differ from the rest, as only 4% of these species with integrons contain multiple integrase genes.

**Figure 1:**
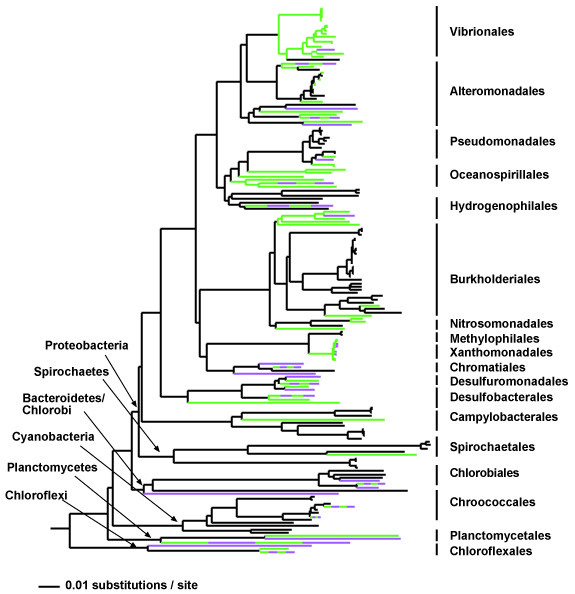
**Phylogenetic distribution of integron- and pseudointegron-containing bacteria. **Lineages colored in green represent integron positive lineages; purple represents lineages with pseudointegrons, and black represents lineages without integrons (organisms which have been entirely sequenced and contain no *intI *gene). Species that contain pseudo and functional integrons are colored both purple and green. 16S rRNA genes from all integron positive (functional and pseudo) and integron negative organisms in the eighteen bacterial orders in which integrons are found were aligned using the NAST aligner [64]. The tree was constructed in PAUP [61] using the NJ algorithm using the best-fit model of sequence evolution calculated in MODELTEST [66]. Orders and major bacterial phyla are indicated. Tree was rooted using five archaeal 16S rRNA gene sequences: *Pyrobaculum clidifontis*, *Aeropyrum pernix*, soil clone cren34kb, *Methanobacterium thermoautotrophicum*, and *Methanosphaera stadtmanae *from the Greengenes [64] alignment.

We mapped the integron phenotype (putative functional genes in green, putative non-functional in purple) onto a 16S rRNA gene tree of all orders of bacteria that contain integrons (again, for simplicity, the typically mobile integrons were excluded). All species reported in Additional File 1 were included in this phylogeny as integron-positive, while all organisms whose genomes are completely sequenced and do not contain *intI *genes were designated integron-negative. The distribution of integron-positive lineages suggests that integron-containing organisms comprise a significant fraction of the phylogenetic diversity within these bacterial orders (Figure 1). Specifically, 67.1% of the total branch length within the 16S rRNA gene tree leads to modern genomes that contain integrons, indicating that less than a third of the phylogenetic diversity falls only within integron-negative lineages. UniFrac analysis [36,37] showed significant phylogenetic clustering (P < 0.001 using unweighted UniFrac significance test and P test through the UniFrac web site), indicating that closely related lineages tend to share integron-positive or integron-negative status more than would be expected by chance.

The current phylogenetic distribution of integrons supports that loss may be an important feature of integron evolution (Figure 1). In some cases this pattern is apparent even within strains of the same species: for example, *Shewanella baltica *OS195 contains two *intI *genes, while *Shewanella baltica *OS155 lacks integrons. Holmes et al. [38] also reported on the spotty nature of integron distribution, finding that two of three isolates of *Pseudomonas stutzeri *contained *intI *genes. Although some of these differences may also be due to horizontal transfer of integrons between closely related species [23], the abundance of pseudogenes suggests that decay and subsequent loss are also important processes. While intra-species differences in gene cassette composition is predicted and often observed [31,32,39,40] due to the inherent integration/excision activity of integrons, these phylogenetically small-scale differences in the presence or absence of integrons is not an expected feature of a non-mobile gene element. Again, the genus *Vibrio *is a notable exception to this pattern, as all species examined to date harbor integrons.

### What is the evolutionary history of intI genes?

**Figure 2:**
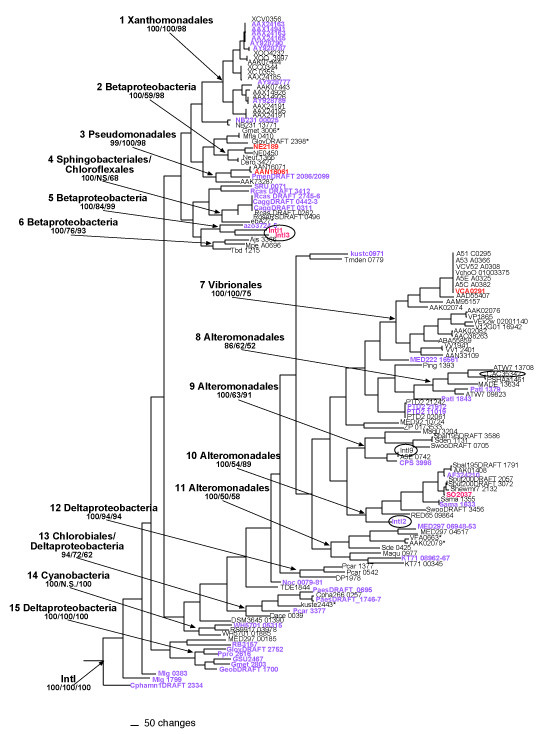
**Representative phylogram showing the relationship between *intI *genes.** Genes colored in purple are putative pseudogenes, sequences in black are putative functional integron integrase genes, and genes colored in red have been shown to be functional *in vivo*. Circled genes are from mobile integrons. IntI proteins were aligned in MAFFT [58] and used to generate a DNA alignment. We used MrModelTest [60] to select the best-fit model of sequence evolution for MrBayes [59]. Rapid likelihood analysis trees where constructed in RAxML-VI-HPC [63]. PHYLIP [62] was used for constructing parsimony trees. Amino acid phylogenies also support the clades indicated (data not shown). The tree shown is the maximum likelihood estimation, and numbers for each clade indicate the maximum likelihood bootstrap, Bayesian posterior probability and maximum parsimony bootstrap values, respectively. The phyla names indicate the dominant organism type found within each clade. The tree was rooted with *xerC *and *xerD *genes from *Escherichia coli *and *Thiobacillus denitrificans*. Gene names and accession numbers are provided in Additional File 1.

We performed extensive phylogenetic analyses using a variety of optimality criteria (maximum likelihood, Bayesian inference, maximum parsimony and minimum evolution) on all *intI *genes and the predicted protein sequences listed in Additional File 1 as well as on the typically mobile *intI *genes (Figure 2). For all methods, *intI *genes formed a supported clade, exclusive of the XerC/XerD outgroup [17,41], with a total of fifteen *intI *clades that were supported by nearly every analysis (Figure 2). Although our tree structure hints at the recently described soil/freshwater and marine groups [17], these clades are not supported by all of our phylogenetic analyses. Each clade of integrase genes primarily originates from organisms within a specific bacterial lineage (clades 1–15, with taxon names in Figure 2; colored boxes in Additional File 1). The vast majority of these *intI *genes have been identified by sequence similarity only; thus their functions remain unknown. However, the seven *intI *genes that have been functionally characterized *in vivo *are widely distributed throughout the *intI *phylogeny (highlighted red in Figure 2), supporting a similar role for the uncharacterized genes in the integration and excision of gene cassettes [20,38,42-45]].

The integrase gene tree suggests some similarities between integron and organismal evolutionary relationships, and in many cases the *intI *genes found within supported clades derive largely from a related group of bacteria (Figure 2). However, there is some evidence for incongruent *intI *and organismal phylogenies, and some of these inconsistencies may be due to horizontal gene transfer events. As stated above, many organisms contain multiple *intI *genes, and in some cases, the *intI *phylogeny suggests that a horizontal gene transfer event may have given rise to one of the genes (Figure 2, Additional File 1). For example, a gene from each of the deltaproteobacterial species *Geobacter metallireducens *and *Geobacter lovleyi *falls just outside of integrase clade 2, which contains genes primarily from within the Betaproteobacteria. These organisms harbor at least two *intI *genes (*G. lovleyi *is not completely sequenced yet and therefore may contain more), but the other genes fall into a supported clade with sequences from the Deltaproteobacteria (clade 15, Figure 2). This is also true for *Shewanella baltica *and *Shewanella woodyi*, which both contain two *intI *genes, one which falls in clade 9 and the other which falls in clade 8.

**Figure 3:**
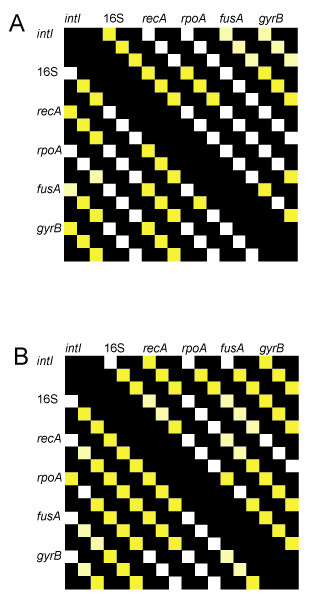
**Likelihood-based tests (KH and SH, [46]) for phylogenetic congruence between *intI *and a variety of housekeeping genes.** Comparisons indicate if there is a significant difference between the fit of the data to trees generated from the different datasets. Boxes above the diagonal represent comparisons using the genes listed above as the reference dataset, while boxes below the diagonal represent comparisons using the genes listed on the side as the reference dataset. The three boxes for each comparison represent distance, parsimony and likelihood based analyses, respectively. White boxes indicate no significant difference, light yellow boxes indicate that one test revealed significant (p < 0.05) differences and yellow boxes indicate that both tests revealed significant differences.

Indeed, *intI *mobility is a character state that is widely distributed across the integrase phylogenetic tree – found in clades 5, 7, 8, and 9 – supporting that the mobile phenotype has arisen multiple times (Figure 2, circled genes) [23]. Additionally, although at first glance, integron integrase genes roughly fall into supported clades reflecting the organisms from which they originated (Figure 2), there are significant differences between integrase and 16S rRNA gene phylogenies [25]. We performed likelihood-based tests for congruence [46] on the *intI *and 16S rRNA gene phylogenies for Deltaproteobacteria (clade 15) and *Shewanella *(clade 10). In all cases, there were significant differences between the two phylogenies (Figure 3). However, these data are somewhat difficult to interpret because in many cases the relationships among several "housekeeping" genes (e.g., *fusA*, *rpoA*, *recA*, *gyrA*) were also determined to be significantly different from the 16S rRNA phylogenies, and from one another (Figure 3). Assuming that the 16S rRNA gene acts as the most accurate metric of genomic evolution, several factors could lead to the lack of concordance between 16S rDNA and integrase relationships. The presence of paralogous *intI *genes, selection, and horizontal gene transfer events could all lead to a lack of agreement between organismal and integrase phylogenies. Paralogous genes are certainly a factor, as many species contain multiple genes, some which appear to have arisen recently within the genome from gene duplication events (Additional File 1, Figure 2). However, even when these events are ignored, and all nodes that are shared between the 16S rRNA gene tree and any paralog are identified, there is a notable lack of concordance. Genetic exchange may also provide a plausible explanation for these patterns, and the association of integrons with transposons and transposase genes provides a possible mechanism for transfer [23]. Finally, differential selection on the 16S rRNA gene and the integrase gene could also contribute to a lack of agreement between phylogenetic reconstructions, and is an interesting possibility given the evidence for positive selection on IntI proteins (see below).

### Pseudogenes

As mentioned above, pseudogenes make up more than one third of all integrase genes uncovered in this study. Others have found *intI *pseudogenes in a number of different bacteria [19,24,39]. For example, the typically mobile class 2 integron integrase, *intI2*, is a non-functional pseudogene, the activity of which can be recovered by suppressing the internal stop codon [19]. Additionally, *intI *pseudogenes were prevalent in a recent survey of integrons from *Xanthomonas *strains [39]. Pseudogenes are also abundant in molecular phylogenetic surveys of *intI *genes from environmental samples, comprising 4–20% of total genes in these collections (Nemergut and Schmidt, unpublished data; Rodriguez-Minguela et al., unpublished data, accession numbers DQ282376–DQ2822194). In general, pseudogenes are relatively rare features of bacterial genomes, as non-functional genes typically comprise only 2–8% of all genes in free-living bacteria [47,48]. Therefore, the high percentage of pseudo-integrase genes, or "pseudo-integrons", that we uncovered is surprising. Recent analyses suggest that pseudogenes are likely to be of contemporary origin, as the same pseudogenes are typically not found in the genomes of closely related bacteria [47,48]. Indeed, orthologs of both functional and non-functional integrases are found in different strains of the same species of *Xanthomonas *[39]. The widespread phylogenetic distribution of pseudointegrons within the bacteria (Additional File 1, Figure 1) implies that decay is a common feature across lineages.

Likewise, the scattered distribution of pseudogenes on the *intI *phylogenetic tree (Figure 2), suggests that gene decay is a frequent process across different classes of integrase genes. For the most part, these appear to be randomly arrayed on the tree, supporting the hypothesis that pseudogenes decay rapidly and are quickly removed from genomes [47,48]. However, integrases within clades 4 and 15 are notable exceptions. Interestingly, despite the minimal evolutionary distance among the pseudogenes found in clade 4, these mutations all appear to have originated independently by unique frameshift mutations. This may be an artifact of biased sampling, or it may suggest that strong purifying selection is acting on genes in clade 4, contributing to their rapid decay. In contrast, a single gene fusion event characterizes an entire group of integrase genes within clade 15, and these proteins are an average of ~120 amino acids longer than other IntI proteins. As mentioned previously, pseudogenes are not generally conserved between closely related species [47,48] as they are typically lost at rates higher than speciation. Thus, the shared gene fusion event within clade 15 may suggest that these are actually functional genes.

Pseudogenes are relatively common features of some eucaryotic genomes, particularly vertebrates [49], where they arise from either retrotransposition or DNA duplication events. In bacteria, duplication processes give rise to pseudogenes as well; however, they are also thought to develop via the decay of native single-copy genes and following failed horizontal transfers [50]. Our phylogenetic analysis suggests that many of the pseudointegrons that we identified appear to have arisen through intragenomic duplication events (a nearly identical functional integrase and pseudogene within the same genome), while others appear to have entered the cell through gene transfer processes (a pseudogene from a different clade in the same genome with a functional gene from the "expected" clade), and still others appear to have been resident genes that are undergoing decay (only one pseudogene from the "expected" clade in the genome) (Figure 2, Additional File 1). In a recent multigenome analysis of bacterial and archaeal pseudogenes, Liu and coworkers revealed that while pseudogenes occur in only approximately 1–5% of total genes, the proportion of integrase pseudogenes are significantly higher, suggesting a different evolutionary dynamic for this class of genes [50]. While, in some cases, the ability to integrate and excise foreign DNA may be selectively advantageous, the gain, loss or rearrangment of gene cassettes could also be deleterious, selecting against functional integrases. Thus, such a high fraction of pseudogenes may suggest that the selective impact of integrons on genomes is variable, oscillating between beneficial and deleterious, possibly depending on environmental conditions. Other interpretations are possible as well, and, as noted by one insightful reviewer, the formation of integrase pseudogenes may result when organisms inhabit environments without gene cassettes, or when recombination processes result in the deletion of gene cassettes that maintain the array (e.g., toxin genes [51]).

Finally, there is mounting evidence that not all sequences which have been identified as pseudogenes are without a biological function. In some organisms, for example, pseudogenes appear to be important in the generation of variation within multigene families, including both antibody and antigen determinants [52,53]. These pseudogenes act as a reservoir of sequence diversity and promote the rapid diversification of gene families through intragenomic recombination events. In many of these cases, genes are under "positive selection" pressures, and mutation (i.e., diversification) is favored. A hallmark of genes undergoing positive selection is an increased ratio of non-synonymous (protein altering) to synonymous (non-protein altering) mutations [54] within groups of closely related genes.

**Figure 4:**
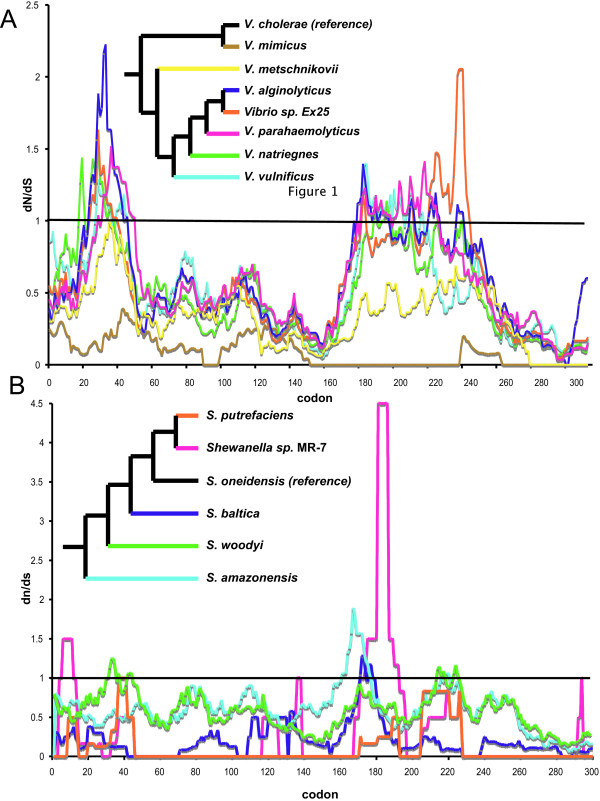
**The ratio of non-synonymous to synonymous mutations over the integrase sequence for *Vibrio *(A) from clade 7 and *Shewanella *(B) from clade 10.** Proteins were aligned in MAFFT [58] and used to generate a DNA alignment. DNA alignments were used to generate pairwise comparisons with the *Vibrio cholerae intI *(A), or *Shewanella oneidensis intI *(B) and the ratio of nonsynonymous and synonymous substitutions at each codon position was calculated. The figure legends show a consensus cladogram (maximum likelihood, maximum parsimony and distance) for the integrase genes.

Thus, we examined the selection dynamics across integrase proteins (Figure 4A). Heuristic dN/dS (also called K_A_/K_S_) pairwise comparisons were made between proteins within integrase clade 7 to the functionally characterized *intI *from *Vibrio cholerae*. This comparison reveals two excursions in the dN/dS ratios above 1, suggesting mutational hotspots ranging from codon ~1 to ~50 and from codon ~160 to ~220. However, when these regions were further examined under the best-fit evolutionary model [54], only the N-terminal domain showed evidence for positive selection (data not shown). This motif is thought to be important in the recognition of attC and attI sites [45], and it is possible that selection for mutation in this region would expand or change the consensus sequences that an integrase is able to recognize. Alternatively, as pointed out by one reviewer, this site is also involved in the expression of gene cassettes [13]; thus, mutation in this region may modulate gene cassette mRNA levels in the host cell. There was a suggestion of a similar pattern for the *Shewanella *proteins in clade 10; however, no pairwise comparisons were significantly greater than 1 when variable evolutionary rates were considered (Figure 4B).

### Characterization of new cassettes

**Figure 5:**
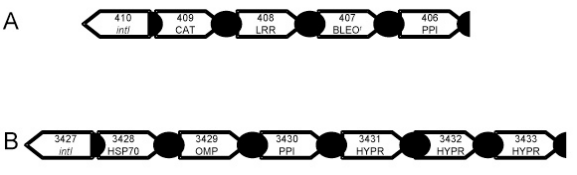
***Methylobacillus flagellatus*****KT (A) and *Dechloromonas aromatica *RCB (B) integrons.** Numbers indicate locus tags; abbreviations: CAT, cation (Co/Zn/Cd) efflux; LRR, leucine rich repeat; BLEO^R^, bleomycin resistance; PPI, c-type peptidyl prolyl cis-trans isomerase; HSP70, heat shock protein 70; OMP, outer membrane protein; HYPR, hypothetical protein.

Finally, we characterized the integron-associated gene cassettes in two species: *Methylobacillus flagellatus *KT, an obligate methylotroph; and *Dechloromonas aromatica *RCB, a perchlorate reducing bacterium (Figure 5). These organisms are found in different orders of the subphylum β-proteobacteria and contain *intI *genes that group with clade 2 (Figure 2). Several of the cassettes that we identified were classified as hypothetical proteins, and pBLAST provided no further clues as to the potential functions of the proteins coded for by these genes. However, several interesting putative functions for gene cassettes were assigned, including a heavy metal (Co, Zn, Cd) pump (CAT) and a heat shock protein (HSP). Additionally, both integron arrays contained genes related to the c-type peptidyl prolyl cis-trans isomerase family, which are involved in protein folding.

## Conclusion

These data reveal several interesting and unusual features of integron evolution, some that appear to be consistent across *intI *and/or organismal clades. First, the present distribution of integrons is likely due to multiple loss events (Figure 1) as well as horizontal gene transfers (Figure 3). Second, many organisms contain multiple, sometimes phylogenetically distinct, integrons (Additional File 1, Figure 2). Third, a high percentage of *intI *genes are non-functional (Additional File 1; Figures 1, 2). Fourth, positive selection seems to be occurring on one IntI domain, at least in the vibrios and perhaps in *Shewanella *(Figure 4). And, finally, *intI *genes form supported clades that roughly reflect the order of the species from which they originated, but in some cases there are obvious exceptions (Figure 2, Additional File 1). Together, these results highlight the dynamic nature of integron evolution, and provide further support that these genetic elements may be important in the genomic fluidity of a large number of diverse bacteria.

## Methods

### Integrase phylogeny

The *Enterobacter cloacae *IntI1 protein sequence (GenBank accession ABO46012) was used to search the nr GenBank database with the PSI-BLAST algorithm and to query the NCBI microbial genome database using the tBLASTn algorithm on April 20, 2007 [55]. All coding region matches that contained the *intI *integrase additional domain [56], or *intI *patch [57] were selected for subsequent analysis, as were the corresponding putative translated sequences. Pseudogenes were identified as those coding regions that were either interrupted by a frameshift mutation or a stop codon, or as proteins that were at least 20% larger or smaller than the *intI1 *gene. For phylogenetic analyses of pseudogenes, we selected only the translated region that aligned to the IntI1 protein and the corresponding DNA sequence. For pseudogenes that contained multiple mutations, alignable regions of both DNA and proteins were concatenated so that their sequences were contiguous. It is of note that NB231_00025, an *intI *sequence from *Nitrococcus mobilis *Nb-231, may not be a pseudogene, as it was found on the end of a shotgun sequence and may be complete within the contiguous genome.

The online version of the sequence alignment program MAFFT [58] was employed to align the amino acid sequences and four outgroup sequences (XerC and XerD from *Escherichia coli *and *Thiobacillus denitrificans *[17,41]) using the following settings: FFT-NS-I, and the BLOSUM45 model. Amino acid alignments were manually adjusted, and any unalignable regions, particularly from within pseudogenes, were trimmed and removed from further analysis. Next, a Python script was developed to guide the insertion of gaps into the corresponding coding DNA sequence for the production of a DNA-based codon alignment (Robeson, unpublished program).

These coding DNA data sets were analyzed using the parallel version of MrBayes [59] and run anywhere from two million to eight million generations in order to achieve convergence under the GTR+I+G model of evolution, as selected via MrModelTest [60]. Consensus trees from the analyses were constructed in PAUP*4.0 [61] after typically removing ~10% of the burnin trees. Parsimony phylogenetic reconstructions were performed using the PHYLIP [62] software package and subjected to 100 bootstrap replicates with 10 randomizations of taxa input order for each bootstrap. Maximum Likelihood analysis used the GTRGAMMA model and was subjected to 100 boostrapped replicates in RAxML-VI-HPC [63].

We performed likelihood-based tests for congruence [46] on the *intI*, 16S rRNA, *fusA*, *rpoA*, *recA*, *gyrA *gene trees for Deltaproteobacteria (clade 15) and *Shewanella *(clade 10). For each comparison, we downloaded DNA sequences from Genbank for all available phyla, and generated trees using distance, parsimony and likelihood optimality criteria. Likelihood tests were executed in PAUP*4.0 as described previously [25].

### 16S rRNA gene phylogeny

For all organisms found to contain integrons, available 16S rRNA gene sequences were obtained from GenBank. We were unable to obtain 16S rRNA gene sequences for all species with integrons; specifically: Alteromonadales bacterium TW-7, *Vibrio sp*. DAT722 [31], *Xanthomonas sp*. CIP 102397 [24], *Vibrio cholerae *MAK 757, *Vibrio cholerae *MZO-3, the *Xanthomonas *DAR strains [39], *Shewanella putrefaciens *CIP 69.34 [24]. In addition, because of the lack of availability, several of the 16S rRNA genes that we obtained were not from the same strain that the integron was identified in, specifically: *Listonella anguillarum*, *Listonella pelagia*, *Vibrio mimicus*, *Vibrio metschnikovii*, *Vibrio natriegens*, *Vibrio salmonicida*, and *Xanthomonas badrii*. All told, we found integrons in eighteen different bacterial orders. 16S rRNA genes from integron containing lineages were aligned with 16S rRNA genes from completely sequenced bacteria from within these same eighteen orders that lacked integrons, as well as with five archaeal 16S rRNA gene outgroup sequences: *Pyrobaculum clidifontis*, *Aeropyrum pernix*, soil clone cren34kb, *Methanobacterium thermoautotrophicum*, and *Methanosphaera stadtmanae*. We used the online NAST-based algorithm available from the Greengenes website [64] to align the 16S rRNA genes, imported alignments into ARB and exported the alignment using lanemaskPH to remove hypervariable regions [65]. MODELTEST [66] was used to estimate the best-fit model of sequence evolution for 16S rRNA gene alignments, and a minimum evolution-based phylogenetic reconstruction was generated in PAUP 4.0. Maximum parsimony and maximum likelihood methods yielded largely similar phylogenic reconstructions but were not shown.

### dN/dS ratios and mutational frequencies

Integrase protein alignments for sequences in clades 7 and 10 were selected for pairwise comparisons to the functionally characterized *Vibrio cholerae *and *Shewanella oneidensis *IntI proteins, respectively. Only putative functional integrase proteins that were >95% different from all others and that originated from within the genus *Vibrio *or *Shewanella *were analyzed. Amino acid alignments were generated using MAFFT with the parameters described above. Next, a codon-based DNA alignment was constructed using our Python script. DNA alignments were then formatted to compare two sequences at a time, using the *Shewanella oneidensis *and *Vibrio cholerae intI *genes as reference sequences. DNA alignments were submitted to the online program SNAP [67] which calculates the change in nonsynonymous (dN) and synonymous (dS) substitutions at each codon position. dN/dS ratios were calculated by integrating over 20 nucleotide positions, and the two-times greater frequency of synonymous sites in a codon was corrected for by dividing this value by 2. dN/dS ratios were plotted against codon position integrated over twenty nucleotide positions. Finally, we used PAML [68] to calculate the dN/dS ratio and likelihood values of a variety of evolutionary models [54].

## Authors' contributions

DRN conceived of the study and drafted the manuscript. MSR carried out the molecular phylogenetic studies. RFK conceived of and carried out the dN/dS ratio comparisons. APM, SKS and RK helped to draft the manuscript.

## Supplementary Material

Additional file 1 Integron integrase genes used in this study. The accession numbers for the genes used for the phylogenetic analyses in this study. Click here for file
