# Supplementary material for: Insights and inferences about integron evolution from genomic data
Source: BMC Genomics. 2008 May 31;9:261. doi: 10.1186/1471-2164-9-261 (PMC2426708; doi:10.1186/1471-2164-9-261)
Supplement: Additional file 1 — Integron integrase genes used in this study. The accession numbers for the genes used for the phylogenetic analyses in this study. [file 1471-2164-9-261-S1.doc]

**Additional file 1.** Integron integrase genes used in this study.

| **Division**  **Class**  **Order** | **Organism**  **(accession)** | **Locus tag/**  **accession** | **Locus tag/**  **accession** | **Locus tag/**  **accession** | **Locus tag/**  **accession** |
| --- | --- | --- | --- | --- | --- |
| Bacteroidetes/ Chlorobi group |  |  |  |  |  |
| Bacteroidetes |  |  |  |  |  |
| Sphingobacteriales |  |  |  |  |  |
|  | *Salinibacter ruber* DSM 13855 (CP000159) whole genome | SRU_0071*^4^ |  |  |  |
| Chlorobia |  |  |  |  |  |
| Chlorobiales |  |  |  |  |  |
|  | *Chlorobium phaeobacteroides* DSM 266 (CP000492) whole genome | Cpha266_0257^13^ |  |  |  |
|  | *Chlorobium phaeobacteroides* BS1 (AAIC00000000) | Cphamn1DRAFT_2334* |  |  |  |
|  | *Prosthecochloris aestuarii* DSM 271 (AAIJ00000000) | PaesDRAFT_1746 / PaesDRAFT_1747*^a 13^ | PaesDRAFT_0695*^13^ |  |  |
| Chloroflexi |  |  |  |  |  |
| Chloroflexi |  |  |  |  |  |
| Chloroflexales |  |  |  |  |  |
|  | *Chloroflexus aggregans* DSM 9485 (AAUI00000000) | CaggDRAFT_0442 / CaggDRAFT_0443*^4^ | CaggDRAFT_0311*^4^ |  |  |
|  | *Roseiflexus castenholzii* DSM 13941 (AAUM00000000) | RcasDRAFT_0262^4^ | RcasDRAFT_3412*^4^ | Btwn RcasDRAFT_2745 /RcasDRAFT_2746*^b 4^ |  |
|  | *Roseiflexus sp.* RS-1  (AAQU00000000) | RoseRSDRAFT_0496^4^ |  |  |  |
| Cyanobacteria |  |  |  |  |  |
| Chroococcales |  |  |  |  |  |
|  | *Synechococcus sp.* RS9917 (AANP00000000) | RS9917_03978^14^ |  |  |  |
|  | *Synechococcus sp.* WH 5701 (AANO00000000) | WH5701_01885^14^ | WH5701_05315*^14^ |  |  |
| Planctomycetes |  |  |  |  |  |
| Planctomycetales |  |  |  |  |  |
|  | *Blastopirellula marina* DSM 3645 (AANZ00000000) | DSM3645_01390 |  |  |  |
|  | *Rhodopirellula baltica* SH 1  (BX119912) whole genome | RB3157* |  |  |  |
|  | Candidatus *Kuenenia stuttgartiensis*  (CT573071; CT573073) | kuste2443^13^ | kustc0971* |  |  |
| Proteobacteria |  |  |  |  |  |
| Betaproteobacteria |  |  |  |  |  |
| Burkholderiales |  |  |  |  |  |
|  | *Methylibium petroleiphilum* PM1  (CP000555) whole genome | Mpe_A0696^6^ |  |  |  |
|  | *Acidovorax sp.* JS42  (CP000539) whole genome | Ajs_3366^6^ |  |  |  |
| Hydrogenophilales |  |  |  |  |  |
|  | *Thiobacillus denitrificans* ATCC 25259 (CP000116) whole genome | Tbd_1215^6^ |  |  |  |
| Methylophilales |  |  |  |  |  |
|  | *Methylobacillus flagellatus* KT (CP000284) whole genome | Mfla_0410 |  |  |  |
| Nitrosomonadales |  |  |  |  |  |
|  | *Nitrosomonas europaea* ATCC 19718 (AL954747) whole genome | NE0450^2^ | NE2189^2^ |  |  |
|  | *Nitrosomonas eutropha* C91  (CP000450) whole genome | Neut_1366^2^ |  |  |  |
| Rhodocyclales |  |  |  |  |  |
|  | *Azoarcus sp.* BH72  (AM406670) whole genome | azo3724 / azo3725*^5^ |  |  |  |
|  | *Azoarcus sp.* EbN1  (CR555306) whole genome | ebA27 |  |  |  |
|  | *Dechloromonas aromatica* RCB (CP000089) whole genome | Daro_3427^2^ |  |  |  |
| Deltaproteobacteria |  |  |  |  |  |
| Desulfobacterales |  |  |  |  |  |
|  | *Desulfotalea psychrophila* LSv54 (CR522870) whole genome | DP1978^12^ |  |  |  |
| Desulfuromonadales |  |  |  |  |  |
|  | *Desulfuromonas acetoxidans* DSM 684 (AAEW00000000) | Dace_0039^13^ |  |  |  |
|  | *Geobacter lovleyi* SZ  (AAVG00000000) | GlovDRAFT_2398 | GlovDRAFT_2752*^15^ |  |  |
|  | *Geobacter metallireducens* GS-15 (CP000149) whole genome | Gmet_3006 | Gmet_2803*^15^ |  |  |
|  | *Geobacter sulfurreducens* PCA (AE017180) whole genome | GSU2467*^15^ |  |  |  |
|  | *Geobacter sp.* FRC-32 (AASH00000000) | GeobDRAFT_1700^15^ |  |  |  |
|  | *Pelobacter carbinolicus* DSM 2380 (CP000142) whole genome | Pcar_0542^12^ | Pcar_1377^12^ | Btwn Pcar_3377 /Pcar_2646*^13^ |  |
|  | *Pelobacter propionicus* DSM 2379 (CP000482) whole genome | Ppro_2616*^15^ |  |  |  |
| Epsilonproteobacteria |  |  |  |  |  |
| Campylobacterales |  |  |  |  |  |
|  | *Thiomicrospira denitrificans* ATCC 33889 (CP000153) whole genome | Tmden_0779 |  |  |  |
| Gammaproteobacteria |  |  |  |  |  |
| Alteromonadales |  |  |  |  |  |
|  | *Alteromonadales* bacterium TW-7 (AAVS00000000) | ATW7_09823^8^ | ATW7_13708^8^ |  |  |
|  | *Alteromonas macleodii* 'Deep ecotype' (AAOD00000000) | MADE_13634^8^ |  |  |  |
|  | *Colwellia psychrerythraea* 34H (CP000083) whole genome | CPS_3998*^9^ |  |  |  |
|  | *Marinobacter aquaeolei* VT8 (CP000514) whole genome | Maqu_0977^11^ | Maqu_3204^9^ |  |  |
|  | *Marinobacter sp.* ELB17 (AAXY00000000) | ZP_01735339 |  |  |  |
|  | *Pseudoalteromonas atlantica* T6c (CP000388) whole genome | Patl_1843* | Btwn Patl_1379/ Patl_1381*^8^ |  |  |
|  | *Pseudoalteromonas haloplanktis* TAC125 (CR954246) whole genome | PSHAa1461^8^ |  |  |  |
|  | *Pseudoalteromonas tunicata* D2 (AAOH00000000) | PTD2_02061 | PTD2_21242 | PTD2_11019 /PTD2_11024* | PTD2_21912/ PTD2_21917* |
|  | *Psychromonas ingrahamii* 37 (CP000510) whole genome | Ping_1393 |  |  |  |
|  | *Saccharophagus degradans* 2-40 (CP000282) whole genome | Sde_0425^11^ |  |  |  |
|  | *Shewanella amazonensis* SB2B (CP000507) whole genome | Sama_1355^10^ | Sama_1633*^10^ |  |  |
|  | *Shewanella baltica* OS195 (AATK00000000) | Sbal195DRAFT_1791^10^ | Sbal195DRAFT_3586^9^ |  |  |
|  | *Shewanella denitrificans* OS217 (CP000302) whole genome | Sden_1131^9^ |  |  |  |
|  | *Shewanella oneidensis* MR-1 (AE014299) whole genome | SO2037^10^ |  |  |  |
|  | *Shewanella putrefaciens* 200 (AAWY00000000) | Sput200DRAFT_3072^10^ | Sput200DRAFT_2057^10^ |  |  |
|  | *Shewanella putrefaciens* CIP 69.34  (AF324211) | AAK01408^10^ | AF324210*^10^ |  |  |
|  | *Shewanella sp.* MR-7 (CP000444) | Shewmr7_2132^10^ |  |  |  |
|  | *Shewanella woodyi* ATCC 51908 (AAUO00000000) | SwooDRAFT_3456^10^ | SwooDRAFT_0705^9^ |  |  |
| Chromatiales |  |  |  |  |  |
|  | *Alkalilimnicola ehrlichei* MLHE-1 (CP000453) whole genome | Mlg_1799* | Mlg_0383* |  |  |
|  | *Nitrococcus mobilis* Nb-231 (AAOF00000000) | NB231_13771 | NB231_00025*^a^ |  |  |
|  | *Nitrosococcus oceani* ATCC 19707 (CP000126) whole genome | Btwn Noc_0079/Noc_0081* |  |  |  |
| Oceanospirillales |  |  |  |  |  |
|  | *Oceanobacter sp.* RED65 (AAQH00000000) | RED65_09864^10^ |  |  |  |
|  | *Oceanospirillum sp.* MED92 (AAOW00000000) | MED92_10724 |  |  |  |
| Pseudomonadales |  |  |  |  |  |
|  | *Pseudomonas alcaligenes*  (AY038186) | AAK73287^3^ |  |  |  |
|  | *Pseudomonas mendocina* (AAUL00000000) | PmenDRAFT_2086/ PmenDRAFT_2099*^3^ |  |  |  |
|  | *Pseudomonas stutzeri B*  (AY129393) | AAN16071^3^ |  |  |  |
|  | *Pseudomonas stutzeri Q*  (AY129392) | AAN16061^3^ |  |  |  |
| Vibrionales |  |  |  |  |  |
|  | *Listonella anguillarum*  (AY126447) | AAM95157^7^ |  |  |  |
|  | *Listonella pelagia*  (AY014401) | AAK02082^7^ |  |  |  |
|  | *Vibrio alginolyticus* 12G01 (AAPS00000000) | V12G01_16942^7^ |  |  |  |
|  | *Vibrio cholerae B33*  (AAWE00000000) | A5E_0325^7^ | A5E_0742^9^ |  |  |
|  | *Vibrio cholerae* MAK 757 (AAUS00000000) | A53_A0366^7^ |  |  |  |
|  | *Vibrio cholerae* MZO-3 (AAUU00000000) | A51_C0295^7^ |  |  |  |
|  | *Vibrio cholerae* NCTC 8457 (AAWD00000000) | A5C_A0382^7^ |  |  |  |
|  | *Vibrio cholerae* O1 biovar eltor str. N16961 (AE003852) whole genome | VCA0291^7^ |  |  |  |
|  | *Vibrio cholerae* O395  (AAKG00000000) | VchoO_01003375^7^ |  |  |  |
|  | *Vibrio cholerae* V52  (AAKJ00000000) | VCV52_A0308^7^ |  |  |  |
|  | *Vibrio fischeri* ES114  (CP000021) | VFA0663^11^ |  |  |  |
|  | *Vibrio fischeri* strain CIP 103206  (AY014400) | AAK02079^11^ |  |  |  |
|  | *Vibrio metschnikovii*  CIP A267  (AY014398) | AAK02074^7^ |  |  |  |
|  | *Vibrio mimicus*  (AF180939) | AAD55407^7^ |  |  |  |
|  | *Vibrio natriegens*  (AY181034) | AAO38263^7^ |  |  |  |
|  | *Vibrio parahaemolyticus* RIMD 2210633 (BA000031) whole genome | VP1865^7^ |  |  |  |
|  | *Vibrio parahaemolyticus* CIP 75.2T  (AY014399) | AAK02076^7^ |  |  |  |
|  | *Vibrio salmonicida*  (AJ277063) | CAC35342^7^ |  |  |  |
|  | *Vibrio sp.* DAT722  (DQ139261) | ABA55859^7^ |  |  |  |
|  | *Vibrio sp.* Ex25  (AAKK00000000) | VEx2w_02001140^7^ |  |  |  |
|  | *Vibrio sp.* MED222  (AAND00000000) | MED222_16561*^7^ |  |  |  |
|  | *Vibrio vulnificus* CIP 75.4  (AF539751) | AAN33109^7^ |  |  |  |
|  | *Vibrio vulnificus* CMCP6  (AE016795) whole genome | VV1_2401^7^ |  |  |  |
|  | *Vibrio vulnificus* YJ016  (BA000037) whole genome | VV1941^7^ |  |  |  |
| Xanthomonadales |  |  |  |  |  |
|  | *Xanthomonas axonopodis* pv. axonopodis DAR34876 (AY928777) | AY928777*^1^ |  |  |  |
|  | *Xanthomonas axonopodis* pv. vesicatoria DAR26930 (AY928788) | AAX14926^1^ |  |  |  |
|  | *Xanthomonas axonopodis* pv. vesicatoria DAR34895 (AY928775) | AAX24163*^1^ |  |  |  |
|  | *Xanthomonas axonopodis* pv. vesicatoria DAR73877 (AY928776) | AAX24165*^1^ |  |  |  |
|  | *Xanthomonas campestris* pv. begoniae DAR54703 (AY928786) | AAX24195^1^ |  |  |  |
|  | *Xanthomonas campestris* pv. begoniae DAR69819 (AY928785) | AAX24191^1^ |  |  |  |
|  | *Xanthomonas campestris* pv. badrii  (AF324482) | AAK07443*^1^ |  |  |  |
|  | *Xanthomonas campestris* pv. campestris  100069T (AF324483) | AAK07444^1^ |  |  |  |
|  | *Xanthomonas campestris* pv. campestris  DAR30538 (AY928784) | AAX24185^1^ |  |  |  |
|  | *Xanthomonas campestris* pv. campestris str. 8004 (CP000050) whole genome | XC_0355^1^ |  |  |  |
|  | *Xanthomonas campestris* pv. campestris str. ATCC 33913 (NC_003902) whole genome | XCC0344^1^ |  |  |  |
|  | *Xanthomonas campestris* pv. oryzae  DAR61713 (AY928794) | AAX14941*^1^ |  |  |  |
|  | *Xanthomonas campestris* pv. oryzae  DAR61714 (AY928789) | AY928789*^1^ |  |  |  |
|  | *Xanthomonas campestris* pv. vesicatoria str. 85-10 (AM039952) whole genome | XCV0356^1^ |  |  |  |
|  | *Xanthomonas campestris* pv. vitians  DAR73878 (AY928790) | AY928790*^1^ |  |  |  |
|  | *Xanthomonas oryzae* pv. oryzae KACC10331 (AE013598) whole genome | XOO4232^1^ |  |  |  |
|  | *Xanthomonas oryzae* pv. oryzae MAFF 311018 (AP008229) whole genome | XOO_3997^1^ |  |  |  |
|  | *Xanthomonas sp.* CIP 102397  (AF324484) | AAK07447*^1^ |  |  |  |
|  | *Xanthomonas translucens* pv. translucens DAR26929 (AY928787) | AY928787*^1^ |  |  |  |
| Unclassified |  |  |  |  |  |
|  | *Congregibacter litoralis* KT71 (AAOA00000000) | KT71_00345^11^ | KT71_08962/ KT71_08967*^11^ |  |  |
|  | *Reinekea sp.* MED297  (AAOE00000000) | MED297_04517^11^ | MED297_00185 | Btwn MED297_06948/ MED297_06953*^11^ |  |
| Spirochaetes |  |  |  |  |  |
| Spirochaetes |  |  |  |  |  |
| Spirochaetales |  |  |  |  |  |
|  | *Treponema denticola* ATCC 35405 (AE017226) whole genome | TDE1844 |  |  |  |

*pseudogene

^a^putative integrase coding sequence includes two open reading frames

^b^putative integrase coding sequence is found between two open reading frames

^c^NB231_00025 was found on the edge of a contig, and may not be a pseudogene.

^1^Xanthomonadales, ^2^Betaproteobacteria, ^3^Pseudomonadales, ^4^Sphingobacteriales/ Chloroflexales, ^5^Betaproteobacteria, ^6^Betaproteobacteria, ^7^Vibrionales, ^8^Alteromonadales, ^9^Alteromonadales, ^10^Alteromonadales, ^11^Alteromonadales, ^12^Deltaproteobacteria, ^13^Chlorobiales/Deltaproteobacteria, ^14^Cyanobacteria, ^15^Deltaproteobacteria.
